# Inflammation scores predict the survival of patients with hepatocellular carcinoma who were treated with transarterial chemoembolization and recombinant human type-5 adenovirus H101

**DOI:** 10.1371/journal.pone.0174769

**Published:** 2017-03-29

**Authors:** Chao-Bin He, Xiao-Jun Lin

**Affiliations:** Department of Hepatobiliary Oncology, Sun Yat-sen University Cancer Center, State Key Laboratory of Oncology in South China, Collaborative Innovation Center for Cancer Medicine, Guangzhou, Guangdong, P.R. China; Chang Gung Memorial Hospital Kaohsiung Branch, TAIWAN

## Abstract

**Background:**

The systemic inflammatory response plays an important role in cancer development and progression. An original inflammation-based staging system for predicting survival in patients undergoing transarterial chemoembolization (TACE) combined with recombinant human type-5 adenovirus H101 is not available. This study aimed to validate the prognostic value of inflammation scores for patients with hepatocellular carcinoma (HCC) who were treated with TACE combined with H101.

**Methods:**

The data from 216 patients with HCC who underwent TACE combined with H101 from January 2007 to July 2015 were retrospectively collected, and the association of the inflammation scores with overall survival (OS) was analyzed. Univariate and multivariate analyses were performed to identify variables associated with OS. The prognostic value of the inflammation scores, including the neutrophil-to-lymphocyte ratio (NLR), platelet-to-lymphocyte ratio (PLR), neutrophil/ platelet-to-lymphocyte ratio (NLR-PLR), modified Glasgow Prognostic Score (mGPS), prognostic nutritional index (PNI), prognostic index (PI), tumor-node-metastasis (TNM), Barcelona Clinic Liver Cancer (BCLC) and Cancer of the Liver Italian Program (CLIP) staging systems were analyzed and compared using the areas under the receiver operating characteristic curves (AUROCs).

**Results:**

The estimated 1-, 2-, and 3-year OS rates were 61.3%, 44.2%, and 40.5% for the entire study cohort, respectively; the median OS was 17 months. According to the multivariate Cox proportional hazards model, the pretreatment NLR, tumor diameter and pretreatment alpha-fetoprotein (AFP) levels were independent predictors of OS. The CLIP score had superior discriminative abilities compared with other staging systems, and the NLR-PLR score consistently displayed a higher AUROC value than the other inflammation-based prognostic scores. The combination of the NLR-PLR and CLIP scores exhibited a superior prognostic ability for OS compared to the NLR-PLR or CLIP scores alone.

**Conclusions:**

The NLR-PLR score is a more powerful predictive system than the other inflammation-based scores for patients with HCC who were treated with TACE and H101. The predictive ability may be improved by utilizing a combination of the NLR-PLR and CLIP scores.

## Introduction

Liver cancer is the most prevalent primary malignant hepatic tumor, particularly in East and Southeast Asia and northern and western Africa. A major type of liver cancer occurring worldwide is hepatocellular carcinoma (HCC), which is the fifth most common cancer in the world and the third leading cause of cancer-related death worldwide [1, 2]. Curative approaches, including surgical resection, liver transplantation and local ablation, are the recommended treatment modalities for only 10%-30% of patients with HCC because of an advanced tumor stage at the time of diagnosis and/or underlying advanced liver cirrhosis [3]. The majority of patients who present portal vein tumor invasion or multiple tumors are diagnosed with intermediate-advanced HCC. Transarterial chemoembolization (TACE), which focuses on delivering chemotherapeutic drugs at the tumor while blocking tumor-feeding arteries, is the most frequently used treatment for these patients [4, 5]. In patients with poor liver function, large tumors and portal vein involvement, TACE is less effective when it is administered for longer than 6 months and is associated with a 2-year survival rate of 24–63% [6]. Moreover, TACE can induce the generation of new tumor cells due to liver ischemia and hypoxia, which can upregulate vascular endothelial growth factor expression and enhance angiogenesis [7, 8]. The poor prognosis of patients with intermediate-advanced HCC who are treated with TACE suggests that new strategies for intermediate-advanced HCC are necessary.

Some genetic abnormalities are present in HCC tumor cells, including the activation of oncogenes [9] and the inactivation of tumor suppressor genes [10]. Mutations or deletions in wild-type p53 are present in nearly 50% of all tumor types and indicate a poorer prognosis for patients with HCC [11]. Gene therapy is a potential curative treatment for cancer. The injection of a recombinant adenovirus carrying p53 (rAd-p53) plays an important role in improving the prognosis of patients with cancer. A combination of TACE and rAd-p53 therapy may have a better effect on delaying the progression of HCC and prolonging the survival of patients with HCC than TACE alone [12–15].

H101, a recombinant human type-5 adenovirus (Ad5), is generated by both an E1B and an E3 gene deletion and infects and kills tumor cells by inducing viral oncolysis [16]. The H101 adenovirus selectively replicates in cancer cells but rarely replicates in normal cells because it lacks E1B to inactivate the active p53 present in normal cells. Thus, H101 cannot replicate and lyse normal cells with active p53 [17]. Moreover, the safety of the adenovirus was improved by deleting a 78.3–85.8-nm gene segment in the E3 region, which encodes the adenovirus death protein [18]. It was proved that H101 was able to efficiently infect and replicate in tumor cell lines in vitro [19] and in vivo [20], leading to observed tumor cell killing and growth inhibition. Some studies have evaluated the efficacy and safety of a transarterial infusion of H101 combined with TACE in patients with intermediate-advanced HCC [21, 22]. Some studies have examined the utility of available staging systems, such as the tumor-node-metastasis (TNM), the Cancer of the Liver Italian Program (CLIP) and the Barcelona Clinic Liver Cancer (BCLC) staging scores, for predicting the survival of patients undergoing TACE. These studies often do not exhibit sufficient precision because of the heterogeneity of the study populations [23, 24]. Patients cannot be easily stratified according to their risk levels, and doctor-patient communication is challenging. In addition, an original staging system for predicting the survival of patients undergoing TACE combined with H101 is not yet available. Thus, patients who would most likely benefit from TACE combined with H101 must be recruited from a heterogeneous population with HCC.

Inflammation plays an extremely important role in the development and progression of many malignancies by participating in the neoplastic process, proliferation and migration [25]. The paradoxical roles of the adaptive (lymphocyte immune cells) and innate leukocytes (circulating neutrophils) in inflammatory processes also function as crucial opposing regulators in the development of cancer [26]. The number of immune cell-like neutrophils has been correlated with increased angiogenesis and/or a poor prognosis, which is partially explained by the upregulation of cyclooxygenase-2 expression or the suppression of an anti-tumor adaptive immune response [27–29]. However, lymphocytes are essential components of tumor defense by killing tumor cells and inhibiting cell proliferation or migration [25, 30]. Additionally, adaptive immune cells, such as B-lymphocytes, CD8+ cytotoxic T-lymphocytes and CD4+ helper T-lymphocytes, play extremely important roles in modulating cancer development by lysing tumor cells [26, 31]. Systemic inflammation is a complex process, and its response is assessed using inflammation indexes. Researchers have expressed increasing interest in the role of systemic inflammation as a predictor of outcomes in patients with HCC. An elevated pretreatment neutrophil-to-lymphocyte ratio (NLR) is confirmed to be associated with poor outcomes in patients with HCC [32]. The platelet-to-lymphocyte ratio (PLR) is also reported to be associated with the outcomes of HCC [33]. The Glasgow Prognostic Score (GPS) serves as an independent marker of a poor prognosis in patients with various stages of HCC disease and different levels of liver function [34]. In the study by Ke et al. [35], the prognostic nutritional index (PNI), which combines albumin and lymphocyte levels, was an independent predictor of poor survival in patients with HCC. The prognostic index (PI) was also shown to be a significant prognostic predictor in patients with other cancers[36]. However, researchers have not yet determined which inflammation score is more accurate at predicting the prognosis of patients with HCC receiving TACE combined with H101 therapy.

The purposes of the present study were to evaluate the prognostic value of inflammation-based prognostic scores, including the NLR, PLR, mGPS, PNI, and PI, to ascertain which prognostic score was a feasible prognostic indicator. Moreover, we wished to establish a prognostic system for patients with HCC undergoing TACE combined with H101.

## Patients and methods

This study was approved by the Institutional Review Board (IRB) of the Sun Yat-sen University Cancer Center. Informed written consent was obtained from all individual participants included in the study. All the patients were informed that their tissues and clinical data would be used to clinical research. They could choose agreement or disagreement about their tissues and clinical data used to clinical research. All procedures performed in studies involving human participants were in accordance with the ethical standards of the institutional and/or national research committee and with the 1964 Helsinki Declaration and its later amendments or comparable ethical standards.

### Patients

Patients with newly diagnosed intermediate-advanced HCC who received TACE as an initial therapy at the Department of Hepatobiliary Oncology (Sun Yat-sen University Cancer Center, Guangzhou, China) between January 2007 and July 2015 were enrolled in this study. Five hundred ninety patients were retrospectively recruited during this period. The survival status was recorded for all patients in this study at least at 1 year after enrollment. This study was approved by the Institutional Review Board (IRB) of the Sun Yat-sen University Cancer Center.

Patients were excluded if they only received TACE therapy without H101 or they received hepatic resection, radio-frequency treatment, liver transplantation or another appropriate treatment for HCC. Additionally, patients who were diagnosed with other primary tumors or lost to follow-up were excluded.

### Treatment procedure

A uniform treatment protocol was performed for each patient. Seldinger’s method was performed through the femoral artery with local anesthesia, as previously reported [37]. Chemotherapeutic agents suspended in lipiodol were injected as selectively as possible into the hepatic segmental artery where the target tumor was located. Conventional chemoembolization was performed by administering 300 mg of carboplatin (Bristol-Myers Squibb, NY, USA). Thereafter, chemolipiodolization was performed using 50 mg of epirubicin (Pharmorubicin, Pfizer, Wuxi, China) and 6 mg of mitomycin (Zhejiang Hisun Pharmaceutical Co. Ltd., Taizhou, China). The dose of lipiodol (Lipiodol Ultra-Fluide; Andre Guerbet Laboratories, France) delivered ranged from 5 mL to 30 mL and was determined based on the tumor location, tumor size, and the number of tumors.

H101 was administered into the hepatic artery supplying the tumor(s) via the catheter before the injection of the chemotherapeutic agents. A total of 1.0 × 10^12^ virus particles in 10 mL of 0.9% sodium chloride was administered. Sterile purified viral lots were produced for human clinical use by Shanghai Sunway Biotech (Shanghai, China), and the titer, sterility, and general safety were tested by the National Institute for the Control of Pharmaceutical and Biological Products (Beijing, China) [38].

### Clinical data collection

All clinical and radiological data were retrieved from the patients’ medical records at the Department of Hepatobiliary Oncology of the Sun Yat-sen University Cancer Center. A hematologic examination and imaging examinations, including sonography, magnetic resonance imaging (MRI), contrast-enhanced dynamic computed tomography (CT), and hepatic arterial angiography, were used in clinical practice to determine HCC prognosis, which was based on the findings of typical radiological features in at least two imaging examinations or histologically confirmed by needle biopsy or by a single positive imaging technique associated with elevated serum alpha-fetoprotein (AFP) levels [39].

Vascular invasion was defined as the presence of a thrombus adjacent to the tumor in the portal system or the hepatic vein system with a vague boundary confirmed by at least two imaging modalities [40]. The clinical and radiological data, including age, gender, white blood cell counts (WBC), platelet counts (PLT), neutrophil counts, lymphocyte counts, AFP levels, alanine transaminase (ALT) levels, aspartate aminotransferase (AST) levels, total bilirubin (TBIL) levels, indirect bilirubin (IBIL) levels, alkaline phosphatase (ALP) levels, albumin (ALB) levels, C-reactive protein (CRP) levels, hepatitis B surface antigen (HbsAg) levels, hepatitis B virus deoxyribonucleic acid (HBV-DNA) load, tumor size, tumor number and vascular invasion, were collected and analyzed. The clinical and radiological data were obtained at the time of diagnosis before the initial TACE was initiated. The inflammation-based prognostic scores, including NLR, PLR, mGPS, PNI and PI, were all entered into our study. The detailed clinical and radiological data were shown in S1 File. When the NLR score was combined with the PLR score, a new inflammation-based score system was generated, which the authors designate the combined neutrophil/platelet-to-lymphocyte ratio (NLR-PLR). A combination of the NLR-PLR and CLIP scores was proposed, which simply added the NLR-PLR score to the CLIP score. All of the inflammation-based prognostic scores determined in this study are described in Table 1.

**Table 1:** Inflammation-based prognostic scores.

Scoring systems	Score
NLR	
Neutrophil count:lymphocyte count < 1.77	0
Neutrophil count:lymphocyte count ≥ 1.77	1
PLR	
Platelet count:lymphocyte count < 94.62	0
Platelet count:lymphocyte count ≥ 94.62	1
Modified Glasgow Prognostic Score (mGPS)	
CRP (≤ 10 mg/L) and ALB (≥ 35 g/L)	0
CRP (≤ 10 mg/L) and ALB (< 35 g/L)	0
CRP (> 10 mg/L) and ALB (≥ 35 g/L)	1
CRP (> 10 mg/L) and ALB (< 35 g/L)	2
PNI	
ALB (g/L) × total lymphocyte count × 10^9^/L ≥ 45	0
ALB (g/L) × total lymphocyte count × 10^9^/L < 45	1
PI	
CRP (≤ 10 mg/L) and WBC (≤ 11 × 10^9^/L)	0
CRP (≤ 10 mg/L) and WBC (> 11 × 10^9^/L)	1
CRP (> 10 mg/L) and WBC (≤ 11 × 10^9^/L)	1
CRP (> 10 mg/L) and WBC (> 11 × 10^9^/L)	2
NLR-PLR	
NLR < 1.77 and PLR < 94.62	0
NLR < 1.77 and PLR ≥ 94.62	1
NLR ≥ 1.77 and PLR < 94.62	1
NLR ≥ 1.77 and PLR ≥ 94.62	2

WBC, white blood cell counts; CRP, C-reactive protein; ALB, albumin

### Follow-up

Patient follow-up and postoperative management were performed according to our established guidelines. The patients were followed at least once every 2 months during the first year and once every 3 months thereafter. An AFP test, liver ultrasonography, computerized tomography scanning, and magnetic resonance imaging, were selectively performed as needed. The OS was defined as the duration (in days) from the date of TACE until death. The last follow-up date was August 31, 2016.

### Statistical analysis

SPSS version 22 (SPSS Inc., Chicago, IL, USA) was used to analyze the data. Continuous data were expressed as the means and ranges, and categorical data were shown as frequencies and proportions. Comparisons between two groups were conducted using Student’s t test for continuous data. The chi-square test and Fisher’s exact test were used to compare the categorical variables.

A univariate analysis was performed to assess the significance of the differences in the clinical or radiological data. A multivariate analysis was performed using the Cox regression model for variables with a significant difference in the univariate analysis. The associated 95% confidence interval (CI) was calculated. The OS was analyzed using the Kaplan-Meier method. Significant differences between the groups were identified using the log-rank test. The survival curves were performed using MedCalc software version 11.4.2.0 (http://www.medcalc.be). A P-value < 0.05 was considered statistically significant.

The analysis of the time-dependent receiver operating characteristic (ROC) curves and comparisons were performed using R software version 3.2.2 (The R Foundation for Statistical Computing, Vienna, Austria. http://www.r-project.org) with the ‘survival ROC’ package and the ‘survival ROC.C’ package.

## Results

### Optimal cut-off values for the variables

The NLR score was calculated by dividing the neutrophil counts by the lymphocyte counts. The PLR score was calculated by dividing the platelet counts by the lymphocyte counts. The optimal cut-off values for the NLR and PLR scores were determined using an analysis of the time-dependent ROC. The NLR and PLR scores were associated with the strongest Youden index for the OS prediction, with cut-off values of 1.77 and 94.62, respectively. The threshold for each clinical and radiological dataset was utilized as the cut-off value for these variables.

### Patient characteristics

**Fig 1:**
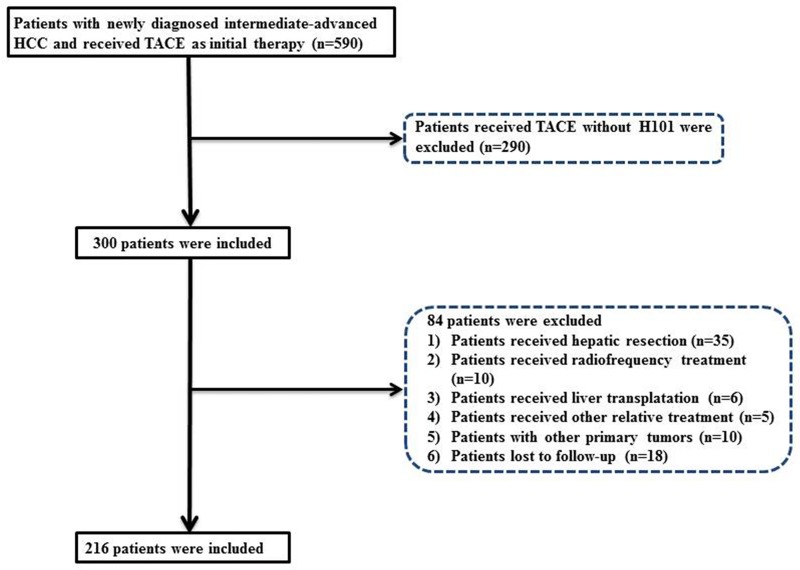
Flowchart of the patient selection process.

Over an 8-year period, between 2007 and 2015, 216 patients who were newly diagnosed with HCC and received TACE combined with H101 treatment as the initial therapy in our hospital were prospectively enrolled and retrospectively analyzed. A flow chart of the selection process for the study cohort is shown in Fig 1.

A total of 200 male (92.6%) and 16 female (7.4%) patients with a median age of 53 years (range: 24–77 years) were enrolled in the study. The majority of the patients had a good reserve liver function with Child-Pugh A. Hepatitis B infection was the most common cause of HCC, and approximately 97.2% of the enrolled patients were positive for HBsAg. Large tumors, for which the diameter exceeded 5 cm, and multiple tumors were more frequently observed in the study cohort. The proportions of patients in the study cohort with large tumors and multiple tumors were greater than 70% and 60%, respectively. In addition, 5.5% of the patients had metastatic tumors, and 30.3% of the patients were sorted into vascular invasion group. The patient demographics and clinical characteristics are detailed in Table 2.

**Table 2:** Clinical and radiological characteristics of the study cohort: Univariate analysis of the factors associated with OS.

Characteristics		N	Value	Univariate	
				HR (95% CI)	P-value
Age (yrs)	< 60/≥ 60	158/58	53.05 (24–77)	0.938 (0.638–1.380)	0.749
Gender	Male/female	200/16	-	1.028 (0.554–1.904)	0.931
WBC (×10^9^/L)	< 10/≥ 10	208/8	6.66 (3–13)	0.458 (0.145–1.442)	0.182
Neutrophil count (×10^9^/L)	< 7/≥ 7	203/13	4.26 (1.3–9.9)	0.750 (0.349–1.612)	0.461
Lymphocyte count (×10^9^/L)	< 0.8 /≥ 0.8	10/206	1.61 (0.2–3.9)	0.508 (0.237–1.088)	0.081
CRP (mg/L)	< 8/≥ 8	98/118	21.32 (0.23–269.43)	2.655 (1.847–3.815)	0.000
PLT (×10^9^/L)	< 100/≥ 100	21/195	194.38 (42–490)	1.161 (0.641–2.103)	0.622
ALT (U/L)	< 40/≥ 40	83/133	60.86 (8–669)	1.281 (0.899–1.826)	0.171
AST (U/L)	< 45/≥ 45	64/152	78.12 (10–871)	2.187 (1.441–3.320)	0.000
ALB (g/L)	< 35/≥ 35	28/188	40.11 (28.7–52.9)	0.617 (0.394–0.969)	0.036
TBIL (mmol/L)	< 20.5/≥ 20.5	169/47	16.45 (5–67)	1.433 (0.966–2.123)	0.073
IBIL (mmol/L)	< 15/≥ 15	199/17	9.67 (3–31.4)	1.285 (0.693–2.382)	0.427
ALP (U/L)	< 100/≥ 100	65/151	160.69 (23.4–1962.8)	1.946 (1.303–2.904)	0.001
GGT (U/L)	< 50/≥ 50	24/192	201.42 (12.2–1433.2)	2.843 (1.389–5.818)	0.004
AFP (ng/mL)	< 400/≥ 400	116/100	20430.51 (1–121000)	2.165 (1.537–3.050)	0.000
HBV-DNA (IU/mL)	< 100/≥ 100	54/162	4073691 (0–2.73×10^8^)	0.967 (0.655–1.427)	0.866
Tumor diameter (cm)	< 5/≥ 5	39/177	9.29 (1.2–22.8)	11.733 (4.334–31.763)	0.000
Tumor number	single/multiple	39/177	-	1.527 (0.949–2.457)	0.081
Vascular invasion	absent/present	133/83	-	2.441 (1.737–3.431)	0.000
NLR	< 1.77/≥ 1.77	49/167	-	3.178 (1.883–5.364)	0.000
PLR	< 94.62/≥ 94.62	75/141	-	1.802 (1.230–2.641)	0.003
mGPS	0/1/2	112/87/17	-	1.977 (1.557–2.510)	0.000
TNM	I/II/IIIa/IIIb/IV	23/4/9/98/82	-	1.868 (1.537–2.270)	0.000
PNI	0/1	44/172	-	0.525 (0.355–0.777)	0.001
PI	0/1/2	112/103/1	-	2.701 (1.928–3.785)	0.000
CLIP	0/1/2/3/4	5/51/60/75/25	-	1.928 (1.601–2.322)	0.000
BCLC	A1/A2/A4/B/C	23/4/9/98/82	-	1.607 (1.315–1.964)	0.000

WBC, white blood cell counts; PLT, platelet counts; ALT, alanine aminotransferase; AST, aspartate aminotransferase; ALB, albumin; TBIL, total serum bilirubin; IBIL, indirect serum bilirubin; ALP, alkaline phosphatase; GGT, gamma-glutamyl transpeptidase; CRP, C-reactive protein; AFP, alpha-fetoprotein level; HBV-DNA, hepatitis B virus deoxyribonucleic acid; NLR, neutrophil-lymphocyte ratio; PLR, platelet-lymphocyte ratio; mGPS, modified Glasgow Prognostic Score; TNM, tumor-node-metastasis; PNI, prognostic nutritional index; PI, prognostic index; CLIP, Cancer of the Liver Italian Program; BCLC, Barcelona Clinic Liver Cancer; HBV infection, hepatitis B virus infection; HBV-DNA, hepatitis B virus deoxyribonucleic acid; HR, hazard ratio; CI, confidence interval

The NLR and PLR scores were divided into two groups: < 1.77 and ≥ 1.77, and < 94.62 and ≥ 94.62, respectively. Among the 216 patients, 167 (77.3%) patients had an elevated NLR score; 141 (65.3%) patients had an elevated PLR score; 104 (48.1%) patients had an mGPS > 0; 44 (20.4%) patients had a PNI ≥ 45; and 104 (48.1%) patients were allocated to PI 1 or 2.

### Correlations between the inflammation-based prognostic scores and OS

**Fig 2:**
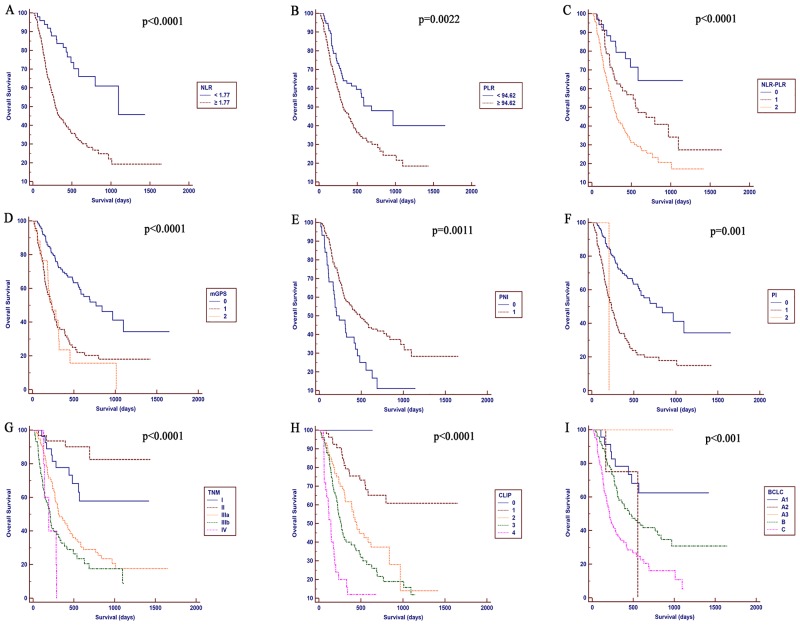
Kaplan-Meier curves for OS in patients with HCC who were treated with TACE combined with H101 were stratified by the inflammation-based prognostic scores and staging system. A, NLR; B, PLR; C, NLR-PLR; D, mGPS; E, PNI; F, PI; G, TNM; H, CLIP; and I, BCLC.

The median follow-up period was 431.11 days (range: 18–1652 days). The estimated 1-, 2-, and 3-year OS rates for the entire cohort were 61.3%, 44.2%, and 40.5%, respectively. The median OS was 17 months. Correlations between the inflammation-based prognostic scores and OS are shown in Fig 1. Elevated NLR (P < 0.0001, Fig 2A), PLR (P = 0.0022, Fig 2B), NLR-PLR (P < 0.0001, Fig 2C), mGPS (P < 0.0001, Fig 2D), PNI (P = 0.0011, Fig 2E) and PI scores (P = 0.001, Fig 2F), as well as higher TNM stages (P < 0.0001, Fig 2G), CLIP scores (P < 0.0001, Fig 2H) and BCLC stage (P < 0.001, Fig 2I) were associated with a reduced OS (all, P < 0.05).

The univariate survival analysis for OS revealed significant associations between an unfavorable OS and higher pretreatment NLR (hazard ratio (HR), 3.178; 95% CI, 1.883–5.364; P < 0.001), PLR (HR, 1.802; 95% CI, 1.230–2.641; P = 0.003), mGPS (HR, 1.977; 95% CI, 1.557–2.510; P < 0.001), PNI (HR, 0.525; 95% CI, 0.355–0.777; P = 0.001), and PI scores (HR, 2.701; 95% CI, 1.928–3.785; P < 0.001), as well as TNM (HR, 1.868; 95% CI, 1.537–2.27; P < 0.001), CLIP (HR, 1.928; 95% CI, 1.601–2.322; P < 0.001) and BCLC stages (HR, 1.607; 95% CI, 1.315–1.964; P < 0.001). Other significant prognostic parameters included the CRP, AST, ALB, ALP, GGT, and AFP levels, the tumor diameter and vascular invasion (Table 2).

According to the multivariate Cox proportional hazards model, the pretreatment NLR score (HR, 2.652; 95% CI, 1.468–4.791; P = 0.001), the tumor diameter (HR, 8.709; 95% CI, 2.964–25.585; P < 0.001) and the pretreatment AFP levels (HR, 1.831; 95% CI, 1.143–2.933; P = 0.012) were independent predictors of OS. Unfortunately, the PLR score failed to be an independent prognostic indicator of OS (HR, 0.890; 95% CI, 0.574–1.379; P = 0.601) (Table 3).

**Table 3:** Multivariate analysis of factors associated with OS based on the study cohort.

Characteristics	Multivariate		
	P-value	HR	95% CI
CRP	0.680	0.851	0.396–1.829
AST	0.961	0.988	0.613–1.592
ALB	0.541	0.790	0.371–1.683
ALP	0.222	1.327	0.842–2.091
GGT	0.625	0.813	0.355–1.864
AFP	0.012	1.831	1.143–2.933
Tumor diameter	0.000	8.709	2.964–25.585
Vascular invasion	0.629	1.146	0.658–1.996
NLR	0.001	2.652	1.468–4.791
PLR	0.601	0.890	0.574–1.379
mGPS	0.369	0.654	0.258–1.652
TNM	0.772	1.065	0.694–1.635
PNI	0.091	0.685	0.441–1.062
PI	0.071	2.693	0.920–7.881
CLIP	0.958	0.991	0.716–1.373
BCLC	0.093	1.410	0.945–2.106

Footnotes are the same as for Table 2.

### Comparative discriminatory performance of the staging systems and inflammation scores

**Fig 3:**
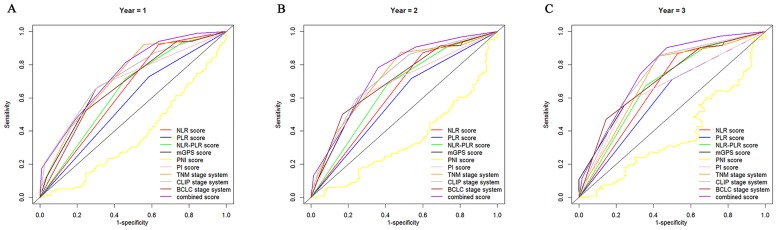
Comparisons of the AUROC values for the survival status between the inflammation-based prognostic scores and the staging systems at 1 (A), 2 (B), and 3-years (C).

The discriminatory capacity of each inflammation score and prognostic system was compared by analyzing the AUROC values. The ROC curves were calculated for the patients’ survival status at the 1-year, 2-year, and 3-year follow-up (Fig 3). As shown in Table 4, the CLIP score had a superior discriminative capacity compared with the other staging systems, and the NLR-PLR score consistently had a higher AUC value than the other inflammation-based prognostic scores.

**Table 4:** Comparison of the AUROC values between the inflammation-based scores and the staging systems.

Score/staging system	Time		
	12 months	24 months	36 months
NLR	0.643	0.637	0.669
PLR	0.572	0.589	0.606
NLR-PLR	0.644	0.653	0.681
mGPS	0.688	0.678	0.673
PNI	0.373	0.347	0.379
PI	0.682	0.675	0.660
TNM	0.722	0.726	0.711
CLIP	0.726	0.731	0.738
BCLC	0.677	0.704	0.713
Combined score	0.744	0.748	0.763

NLR-PLR, neutrophil/platelet-lymphocyte ratio

Other footnotes are the same as for Table 2.

### Prognostic value of the combination of the NLR-PLR and CLIP scores for patients with HCC who were treated with TACE combined with H101

**Fig 4:**
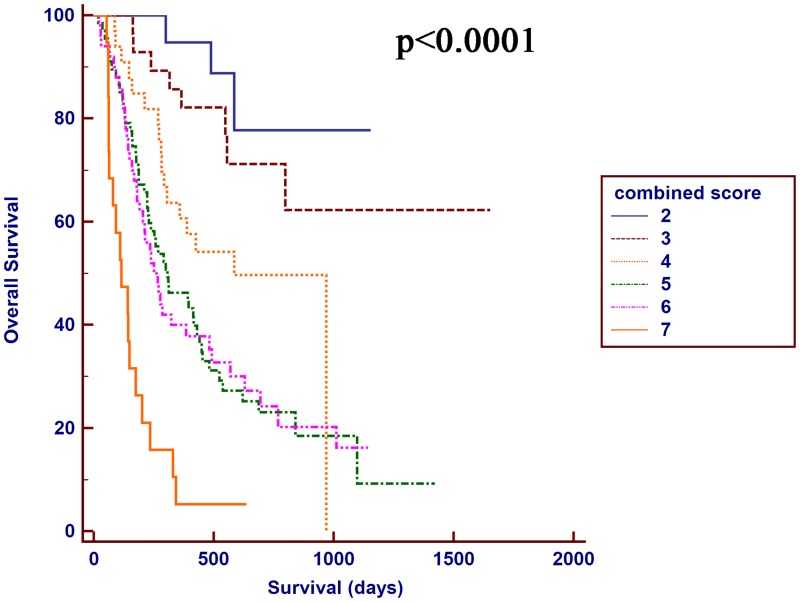
Kaplan-Meier OS curves for the patients with HCC who were treated with TACE combined with H101 stratified by the combined score.

The NLR-PLR and CLIP scores showed a better distinguishing power for predicting the prognosis of patients with HCC who were treated with TACE combined with H101 compared with the other relative score systems. Hence, a combination of the NLR-PLR and CLIP scores was proposed, and the authors designated this score the combined score system. The AUROC value of this combined score was higher than the NLR-PLR score or CLIP score alone. The OS was significantly different between the two groups stratified by the combined score (P < 0.0001, Fig 4). The combined score divided patients into subgroups more precisely.

## Discussion

In terms of the management of the HCC guidelines, TACE provides survival benefits in patients with intermediate-advanced HCC [41, 42]. However, some residual tumor cells persist, resulting in tumor progression shortly after TACE [43]. A modification to TACE is needed for patients with intermediate-advanced HCC. H101 is an adenovirus that is a hybrid of serotypes 2 and 5 and contains a genomic deletion in the E1B and E3 region that results in the loss of expression of the viral 55-kDa protein (E1B 55K), which blocks p53-mediated transcriptional activation [44]. H101 has been shown to replicate independently in many tumor cell lines [45, 46]. The combination of TACE with H101 is a novel therapy that enhances the efficacy of the HCC treatment. A prognostic score must be established to recruit patients who would most likely benefit from TACE combined with H101.

The host systemic inflammatory response plays an important role in cancer development and progression [47]. Growth and survival factors released from inflammatory cells stimulate tumor formation, progression, angiogenesis, invasion and metastasis [48, 49]. Moreover, elevated scores are more frequently observed in patients with vascular invasion, a larger tumor size, and tumors of advanced clinical stages, suggesting that a more aggressive clinical phenotype can be predicted by the systemic inflammatory response [50]. Inflammation-based scores are associated with poor survival in patients with various cancers. However, few reports compare the inflammation scores of patients with HCC, and a consensus regarding the most suitable prognostic score for patients receiving a TACE combined with H101 treatment has not been reached.

The prognostic power for all inflammation-based prognostic scores and staging systems in patients treated with TACE combined with H101 was compared in our study. While clear different prognostic strata were observed for the patients stratified by all the prognostic scores and staging systems, some overlap of the Kaplan-Meier survival curves was observed for the mGPS and PI. The OS of patients was not precisely distinguished by the NLR, PLR and PNI scores. The NLR-PLR score was superior to other inflammation-based prognostic scores. In addition, the prognostic power of the combined score system was greatly improved compared with the other prognostic systems. Although all inflammation-based scores were associated with OS in the univariate analysis in our study, the comparison of the inflammation scores indicated that the NLR-PLR consistently exhibited a higher AUROC value at 1, 2 and 3 years compared with the other scores and showed an excellent discriminatory performance for the patients. These results were also consistent with the findings from another study [51], in which the NLR-PLR score predicted the survival of patients with hepatitis B virus-related HCC within the Milan criteria after liver resection.

The ability of three widely utilized staging systems to predict survival in patients receiving TACE combined with H101 was evaluated, and the AUROC values of the CLIP scores were consistently higher than the TNM and BCLC staging systems. Similarly, Hau CY et al. [52] compared five currently used prognostic models in 1,713 prospectively enrolled patients with HCC and showed that the CLIP staging system was the best long-term prognostic model for patients with HCC in a cohort with early to advanced stages of HCC, and the predictive accuracy of this system was independent of the treatment strategy.

Cancer-related inflammatory responses affect cellular proliferation, cell survival, angiogenesis, tumor cell migration, invasion, metastasis and the inhibition of adaptive immunity, indicating that cancer and inflammation are closely associated [30]. Although our study showed that these prognostic scores or staging systems stratified the patients with HCC who were treated with TACE combined with H101 into distinct risk categories, these systems or scores were suboptimal at predicting OS. According to the Kaplan-Meier survival curves, the NLR-PLR score and CLIP score divided the patients into subgroups more accurately. The combined score exhibited a higher AUROC value than the NLR-PLR score or the CLIP score alone. The Kaplan-Meier survival curves were separated more effectively when the combined score was used to stratify the patients. The combined results of the AUROC and the Kaplan-Meier survival curves were considered an accurate tool to predict the OS of patients with HCC who were treated with TACE combined with H101. In our study, the proportions of patients with large tumors and multiple tumors were greater than 70% and 60%, respectively, and 30.3% of the patients were sorted into the vascular invasion group, indicating that most of our enrolled patients had advanced stage HCC. Some patients with advanced stage HCC do not benefit from TACE combined with H101. The choice of treatment is somewhat controversial because of the lack of a valid prognostic model that clinicians can use to assess patient survival and prescribe the proper therapy. The combined score performed well in predicting the OS for patients with HCC who were treated with TACE combined with H101. Clinicians may be required to choose other treatment strategies for patients with a combined score > 5, which indicated a poor prognosis. Thus, our combined score may provide evidence for individualized treatment in clinical practice.

An elevated AFP level and a larger tumor were also associated with a poor OS in our study, consistent with previous studies [53], and these values were included into the clinical staging system [54, 55].

Our novel combined score had unique features. The combined score, in which the laboratory factors and the radiological factors were both considered, was more powerful and accurate in predicting the prognosis of patients with HCC receiving TACE combined with H101 than the NLR-PLR score and CLIP staging system, which were superior to other inflammation-based prognostic scores or staging systems. This combined score, which includes the most informative factors for survival, is a predictive tool that may be used for patients with HCC receiving TACE combined with H101 at their initial presentation and diagnosis in clinical practice.

This study has some limitations. First, the prognostic factors were restricted to common clinical or radiological data, and some potential biomarkers used to predict HCC and time-dependent variables during the follow-up period were not included in this combined score model. Second, the combined score was generated from patients with HCC receiving the TACE combined with H101 treatment. This score was probably not suitable for patients with HCC receiving other treatments. Third, approximately 97.2% of the enrolled patients were infected with HBV. The geographic and institutional heterogeneity observed among the patients afflicted with HCC could affect the results. This retrospective analysis relied on data from patients with HCC receiving the TACE combined with H101 therapy at a single institution. A large-scale prospective validation study and relative laboratorial experiments are therefore needed to confirm the results and identify more predictive factors.

## Supporting information

S1 File Detailed description of clinical and radiological data. (XLS)
